# Proceedings from the inaugural conference on the science of cancer health equity for Sexual and Gender Minority (SGM) communities

**DOI:** 10.1186/s12919-026-00360-w

**Published:** 2026-02-03

**Authors:** Charles S. Kamen, Milena E. Insalaco, Richard Brown, Chasity Burrows Walters, Shine Chang, Viktor Clark, Jason Domogauer, Lauren V. Ghazal, Kelly Haviland, Forrest Hosea, Nelson Sanchez, Matthew B. Schabath, Zul Surani, Gwendolyn P. Quinn

**Affiliations:** 1 Saunders Research Building, University of Rochester, 265 Crittenden Blvd, Box 420658, Rochester, NY 14642 USA; 2 Virginia Commonwealth University, Richmond, VA USA; 3 Memorial Sloan Kettering Cancer Center, New York, NY USA; 4 The University of Texas MD Anderson Cancer Center, Houston, TX USA; 5 New York University Perlmutter Cancer Institute, New York, NY USA; 6 New York Cancer and Blood Specialists, New York, NY USA; 7 LGBTQ+ Community Advisory Board, New York, USA; 8 H. Lee Moffitt Cancer Center & Research Institute, Tampa, FL USA; 9 Cedars-Sinai Cancer Center, Los Angeles, CA USA

**Keywords:** Cancer, Health Disparities, Sexual and Gender Minorities

## Abstract

**Purpose:**

Sexual and gender minority people (SGM) experience multiple cancer-related disparities, including higher rates of cancer risk factors, lower rates of cancer screening, higher lifetime risk of cancer, and unmet needs throughout cancer survivorship. Although many national organizations, including the National Cancer Institute (NCI) and the American Cancer Society (ACS), have emphasized the need for more cancer research among SGM communities, currently no consensus exists about the highest research priorities, promising research models, or mechanisms to support collaboration between geographically dispersed scientific teams.

**Methods:**

To address this gap, we convened a three-day conference to focus on the “Science of Cancer Health Equity for SGM Communities.” Held at the New York University (NYU) Langone Medical Center Campus in New York City from October 5–7, 2023, this conference brought together researchers, trainees, early-stage investigators, and community members. The conference aimed to: 1) examine the current span of evidence-based research on SGM cancer across the cancer control continuum; 2) establish SGM cancer research priorities; 3) promote the careers and research of trainees and early stage investigators, especially those from minoritized backgrounds; 4) develop a transdisciplinary network of professionals with a focus on mentorship and shared research methods; and 5) disseminate findings from this conference, including priorities for SGM cancer health equity research.

**Results:**

Response to the conference was overwhelmingly positive.

**Conclusion:**

The success of this inaugural conference indicated the need for additional efforts to advance SGM cancer research and expand on scientific priorities.

## Introduction

Sexual and gender minority people (SGM) comprise at least 9.3% of the U.S. adult population and experience high rates of psychological distress and substance use, low rates of insurance coverage, and difficulty accessing responsive and tailored healthcare services [1–7]. These disparities may be due in part to chronic stress arising from discrimination based on sexual orientation and/or gender identity (SOGI), known as minority stress [8–11]. SGM people encounter, witness, or hear about discrimination based on SOGI, which leads them to expect discrimination and experience psychological distress [12, 13]. To the extent that SGM people can develop a sense of social safety through strong connection and belongingness with others, they may be buffered from minority stress [14]. In addition, SGM identities cut across other stigmatized identities, described as intersectional disenfranchisement [15]. Examples of intersecting marginalized identities include SGM people of color [16], those who are economically disadvantaged [17, 18], and those with disabilities [19], all of whom experience more discrimination in healthcare than those without these identities [9]. The importance of addressing discrimination and disparities among SGM people is underscored by designation of sexual and gender minorities in 2016 as a health disparity population by the National Institutes of Minority Health and Health Disparities (NIMHD) and a priority population by the National Cancer Institute (NCI) [20, 21].

Studies focusing on SGM communities across the cancer continuum reveal notable issues and disparities [22, 23]. SGM people experience higher rates of cancer risk factors, including tobacco and alcohol use [6, 7, 13]. Some SGM people may be less likely to receive guideline-based cancer screening [9, 10], and this is especially true for transgender and other gender diverse people [15]. In terms of cancer incidence, an estimated 687,800 to 1,285,100 SGM people are living with cancer in the United States, a number comparable to other priority groups whose health experiences have been the focus of multiple scientific conferences and funding initiatives [24–26]. Cancer rates are higher among some SGM subgroups than the population at large, with gay men and bisexual women at particularly high risk [27–29]. Finally, SGM cancer patients experience significant health disparities, including higher rates of psychological distress, risk for cancer recurrence, and tobacco use after cancer treatment [28, 30–34].

While prior efforts have been made to characterize and address cancer disparities for SGM communities, oncology researchers, clinicians, and SGM community organizations have not yet come together to determine a collaborative path forward [21]. Transdisciplinary perspectives, which holistically span multiple disciplines, are necessary to chart this path, as the cancer-related health disparities facing SGM people cut across individual, social, and systemic domains [35, 36]. To address these disparities, input is needed from oncologists, nurses, epidemiologists, psychologists, and specialists from other academic and clinical disciplines, as well as from SGM-focused community organizations and SGM people with a lived experience of cancer [36]. Policy knowledge and planning are also essential, given that legislative actions that impact SGM rights and protections broadly may have a specific impact on health, well-being, and access to cancer care [37]. SGM cancer health equity research priorities must be set with input from all of these perspectives.

Accordingly, researchers, clinicians, and patient advocates representing seven cancer centers from across the United States came together to organize an inaugural conference on the Science of Cancer Health Equity for Sexual and Gender Minority Communities. The conference was organized to: 1) examine the current span of evidence-based research on SGM cancer across the cancer control continuum; 2) establish SGM cancer research priorities; 3) promote the careers and research of trainees and early stage investigators, especially those from minoritized backgrounds; 4) develop a transdisciplinary network of professionals with a focus on mentorship and shared research methods; and 5) disseminate findings from this conference, including priorities for SGM cancer health equity research.

## Methods

The Science of Cancer Health Equity for SGM Communities conference was held October 5–7.^th^, 2023 at the New York University (NYU) Grossman School of Medicine. The conference was co-led by Dr. Charles Kamen, University of Rochester, and Dr. Gwendolyn Quinn, NYU. The organizing committee consisted of Dr. Richard Brown, Virginia Commonwealth University; Dr. Shine Chang, The University of Texas MD Anderson Cancer Center; Dr. Viktor Clark, Virginia Commonwealth University; Dr. Jason Domogauer, NYU; Dr. Kelly Haviland, Memorial Sloan Kettering Cancer Center (MSKCC); Forrest Hosea, LGBTQ Community Advisory Board; Dr. Nelson Sanchez, MSKCC; Dr. Matthew Schabath, Moffitt Cancer Center; and Dr. Chasity Burrows Walters, MSKCC. The conference was sponsored by a conference support grant (R13 CA190529) from multiple institutes at the National Institute of Health and supported by contributions from the University of Rochester, Virginia Commonwealth University, Columbia University, and Fox Chase Cancer Center and educational grants from Genentech and Bristol Meyers-Squibb. Presentations at the conference included both invited research experts, community organizations, funding agencies, top scoring abstracts and posters. See Table 1 for the agenda; abstracts have previously been published [38].

**Table 1:** Agenda for the inaugural conference on the Science of Cancer Health Equity for Sexual and Gender Minority Communities

**2023**	**Thursday, 10/5**	**Friday, 10/6**	**Saturday, 10/7**
*8:00 AM*	Coffee/registration	Coffee/Poster session #2	Coffee/Poster session #3
*9:00 AM*	Opening Plenary	Day 2 Plenary	Patient and Community Panel
*10:00 AM*	Meet the Funders	Representation in Research	Provocative Questions #3
*10:30 AM*			
*11:30 AM*	Research Models	SOGI Supplement Awardees	Break-Out Reports Wrap Up
*12:00 PM*			
*1:00 PM*	Networking lunch	Networking lunch	Grab-and-go lunch
*2:00 PM*	Funded Researchers	Provocative Questions #2	Organizing Committee Debrief (by invitation)
*3:30 PM*	Provocative Questions #1	Break-Out Sessions	
*5:00 PM*	Opening reception/Poster session #1	GAYLa Panel: "Cancer is a Drag"	

### Day 1 – Thursday Oct 5, 2023

#### Opening plenary

The first day of the conference began with an opening address by Dr. Kamen who then moderated the welcome address by Dr. Abraham Chachoua, deputy director of Perlmutter Cancer Center at NYU. The keynote speaker, Dr. Scout, executive director of the National LGBT Cancer Network, then spoke about the “Decade of SGM Cancer Health Equity Research,” harkening back to a 2014 summit also held in New York City and the progress that had been made in SGM cancer health equity in an almost 10-year period [39].

#### Meet the funders

A panel of representatives spoke on behalf of funding agencies including the National Institutes of Health (NIH) Sexual and Gender Minority Research Office, National Cancer Institute (NCI) Division of Cancer Control and Population Sciences, and the American Cancer Society (ACS). They discussed funding opportunities for research on cancer prevention, treatment, and survivorship in SGM populations. Panelists provided overviews of their agencies, highlighting opportunities in SGM cancer and disparities research, as well as individual program directors for contact. A moderated discussion included guidance about how to interact with program directors successfully, the roles of program officers as stewards of funding and champions for researchers, and how to support funding for research, including federal funding. The moderator called on audience members to identify at least three partners for future research collaboration over the days of the conference and to meet with the representatives from the funding agencies, explaining how developing such relationships could be beneficial to researchers for years.

#### Research models

An invited panel of SGM researchers, which included Dr. Ilan Meyer from the Williams Institute, Dr. Lisa Diamond from the University of Utah, and Dr. Tonia Poteat from the University of North Carolina (now Duke University), addressed the need for theoretical frameworks to guide SGM research. Panelists spoke about research using minority stress, lack of social safety, and intersectional theories, respectively, for SGM cancer health equity research. The speakers offered unique insights into new frameworks for scaffolding SGM research as well as ways to consider existing models in new light. Dr. Gwendolyn Quinn summarized the models shared and moderated a discussion between panelists. The question-and-answer session afterwards featured questions from junior researchers about incorporating these models in their future grant applications, and from senior researchers who were contemplating models for including SOGI data in their existing research.

#### Funded researchers

An invited panel of funded researchers, moderated by Dr. Nelson Sanchez, discussed their work to improve care across the cancer continuum among SGM people. Dr. Ulrike Boehmer from Boston University School of Public Health detailed historical barriers to SGM cancer research and challenged attendees to expand SOGI data collection, develop new research frameworks beyond health disparities models, and propose new comparisons beyond the SGM and non-SGM dichotomy. Dr. Simon Rosser from the University of Minnesota School of Public Health discussed structural changes the National Institutes of Health (NIH) could implement to improve R01 funding for SGM cancer research. Finally, Dr. Phoenix Matthews from Columbia University School of Nursing reviewed best practices in grantsmanship to increase the number of funded SGM cancer R01 applications. Discussion after these presentations centered on improving advocacy for SGM research and strategies for obtaining funding as a new researcher interested in SGM cancer health equity.

#### Provocative questions 1

##### Structural/Systemic Approaches to SGM Cancer Equity

Speakers were selected for a provocative questions symposium based on abstracts they submitted to the conference organizing committee. This symposium was moderated by Dr. Matthew Schabath. The overall symposium theme focused on structural and systemic strategies to improve clinician communication and training for SGM populations with cancer and collecting SOGI data. This session began with Dr. Smita Banerjee from MSKCC on communication skills training for oncology clinicians. Next, Dr. Debra Rodrigue from MSKCC presented on the development of an educational program to improve nurse communication in the care of transgender patients with cancer. Dr. Jane Ussher, from Western Sydney University, was next and presented on the impact of cancer on transgender embodiment and identity. The next presenter was Dr. Megan Mullins from UT Southwestern who presented barriers, facilitators, and a multilevel implementation strategy for routine SOGI collection in community oncology practices. Dr. Benjamin Schrank from MD Anderson Cancer Center presented on piloting the challenges and opportunities in SOGI collection and staff training to collect and use SOGI. And the final presentation from this session was by Dr. Theresa Hastert from Karmanos Cancer Institute who presented on a community-driven adaptation of a cancer caregiving intervention for SGM populations.

### Day 2—Friday Oct 6, 2023

#### Plenary

Day two began with a poster session on clinical care followed by a keynote address by Asa Radix, MD. Dr. Radix spoke on advancing health equity in cancer care for transgender and gender diverse individuals. The talk contextualized research on transgender health in the context of cancer within the broader scope of research on transgender health generally. Dr. Radix also shared the work of the World Professional Association for Transgender Health (WPATH), including a review of transgender health research that highlighted the lack of studies focused on cancer in transgender populations.

#### Representation in research

A research panel of invited presenters reviewed ways that SGM people have been represented in cancer research. Dr. Kellan Baker shared the work of the National Academies of Sciences, Engineering, and Medicine (SOGI) to standardize items for SOGI data collection. Other presenters shared work to adapt the NASEM SOGI items for the context of cancer and qualitative research with the transgender community on their preferred approach to SOGI data collection. The question-and-answer session, moderated by Dr. Burrows Walters and Forrest Hosea, focused on ways to adapt SOGI items to new contexts and communities, as well as safety and protection for those disclosing SOGI.

#### Sexual Orientation and Gender Identity (SOGI) data collection supplement awardees

Following the invited presentations on SOGI data collection were brief presentations by 12 of the 13 institutions that received supplements to their NCI P30 Cancer Center Support Grant awards to implement SOGI data collection. Representatives from Yale University, University of Southern California, NYU, Moffitt Cancer Center, Baylor College of Medicine, Thomas Jefferson University, Wayne State University, Roswell Park Cancer Institute, Northwestern University, Ohio State University, and University of New Mexico, and University of Pittsburgh all shared progress on their implementation studies, which ranged from qualitative data collection approaches to efforts to integrate with electronic medical records.

During the lunch hour, representatives from Genentech shared how they were approaching SOGI data collection from an industry perspective.

#### Provocative questions symposium 2: Pediatric/AYA Issues in SGM communities

After lunch another symposium, comprising six speakers chosen from the top scoring abstracts, presented on a second set of provocative questions focused on pediatric, adolescent, and young adult SGM cancer survivors. The presentations included discussion on the impact of cancer on quality of life and hardships. This included Dr. Janette Perz from Western Sydney University on SGM cancer patients’ quality of life and distress, as well as Austin Waters from the University of North Carolina on financial hardship differences. The additional presentations focused on discussions surrounding collection and inclusion of AYA patient sexual orientation and gender identity along the cancer continuum. These included Dr. Nina Jackson Levin from University of Michigan on AYA cancer patients’ experiences with disclosing SOGI, Dr. Madeline Harper-Bono of Rutgers University exploring pediatric cancer providers’ presence of online inclusive care for SGM AYA patients, Dr. Brad Zebrack on inclusive design of clinical trial and recruitment strategies for SGM AYA cancer survivors, and Dr. Lauren Ghazal from the University of Rochester and Hailey Johnston from the University of Michigan discussing a needs assessment approach for AYA SGM cancer survivors.

#### GAYLa panel on breast/chest cancer

An evening event, Cancer is a Drag, was held at Memorial Sloan Kettering Cancer Center. As part of the event, sponsored by the Evelyn H. Lauder Breast Center, Dr. Haviland moderated a panel discussion about breast cancer in the SGM community with panelists Dr. Neil Iyengar, Yee Won Chong, and Nyx Melody. The discussion revolved around the unmet needs of breast cancer in SGM patients including prevention, screening, treatment, and survivorship.

### Day 3 – Saturday Oct 7, 2023

#### Patient and community panel

The third day of the conference began with a patient and community advocate panel presentation. The patient panel consisted of Jessica Brescher, MPH, Paula D. Chambers, Darryl Mitteldorf, MSW, eMPA, Alexandr Trifonov, and Bennie B. Gross, BSN, RN and was moderated by Drs. Clark and Kamen. During this panel discussion, patient advocates such as Paula Chambers, Alexandr Trifonov, and Bennie Gross described the need for shared decision-making, affirmation of SGM identities, and community collaborations to improve the current state of cancer disparities among SGM individuals. Jessica Brescher, Darryl Mitteldorf, and Bennie Gross described the importance of SOGI data collection to improve tailored healthcare. However, the barriers to SOGI data collection such as differences across electronic medical record software to uniformly collect SOGI data, medical mistrust, and SGM stigma across the country all exacerbated SGM cancer inequities. Overall, this panel urged the audience to engage with SGM communities to gain insight into community members’ expert opinions on barriers and facilitators to cancer-related outcomes across the country.

#### Provocative questions symposium 3: SGM cancer screenings and sexual health

Afterwards a final symposium of 6 speakers, representing the top scoring abstracts, presented on a third symposium of provocative questions focused on cancer screenings and sexual health. Dr. Irene Tami-Maury reviewed an R25 training program designed to build capacity for an SGM focused cancer research agenda. Participants reported greater knowledge about cancer care. Screening, a vital component of cancer care was addressed by Dr. Pheonix Matthews who spoke about tobacco use among SGM Populations, addressing factors that account for higher rates of smoking and targeted interventions to address inequities in screening. Josie Raphaelito continued the theme addressing colon cancer screening in Two Spirit and Native American SGM populations. Josie described the adaptation of an existing intervention that has potential to address inequities in screening in Two Spirit and Native SGM. Further evidence for the importance of screening was provided by Dr. Michael Ross who detailed the increased risk of Oropharyngeal cancer in MSM. Simply taking an oral selfie is an important tool in increasing detection. Dr. Alan Nitray added the need for HPV vaccination and screening to decrease rates of anal cancer in people living with HIV. Dr. Christopher Weldon turned attention to a different aspect of cancer care: the use of anal dilators to decrease problems in sexual functioning in anal receptive sex. The study conclusion showed a need to increase engagement with anal dilators and further test efficacy.

#### Break-out sessions and key takeaways

To gather feedback from attendees on priorities for future SGM cancer research, attendees split into three breakout sessions: one session was on clinical care, another was on supportive care, and the last was on epidemiology and public health. In the clinical care breakout session, building trust with clinicians and patient autonomy were found to be main priorities. Some actions discussed to help foster trust and autonomy were empowering patients to engage in shared decision making about their cancer treatment, having clinicians connect with community members in non-clinical community spaces, having clinicians connect patients with other SGM competent clinicians and resources, and establishing opportunities for SGM people to give their feedback on their experiences. In the supportive care breakout session, the main priorities were overcoming barriers to fostering diversity and intersectionality. The concept of safety was discussed in respect to the history of medical mistrust due, fear due to the COVID-19 pandemic, and anxieties due to the current political climate. Attendees stated there was also an inadequate amount of diverse SGM representation due to a lack of funding for SOGI related projects, a lack of diversity on editorial boards, and lack of diversity of SGM identities, as many studies group all sexual and gender minorities together. In the epidemiology and public health breakout session, people discussed the importance of going beyond including intersectional identities to use methodologies that can effectively evaluate intersectionality in participants and communities. Two specific projects were discussed in this group: 1) a longitudinal study of transgender patients undergoing treatment, and 2) a task force with SEER to collect SOGI data.

Feedback about the conference was overwhelmingly positive, with attendees sharing, “I was moved, thrilled, educated, and overwhelmed,” “This conference has left me inspired to continue pursuing SGM cancer health equity research,” “This conference was an energizing experience that makes me hopeful about the coming growth and improvement in SGM cancer care, “I benefitted enormously from the networking opportunities made available through this conference,” and “The event is necessary and should continue as an annual conference.”

Based on this feedback and interest from others in the field, the conference organizers agreed to plan additional conferences in subsequent years to continue to refine priorities for SGM cancer research and give scientists and trainees in the field opportunities to network. These proceedings can form a template that could be used for other organizers hoping to engage transdisciplinary researchers to address a problem such as health disparities, or to convene a group of interest-holders interested in a specific and circumscribed topic. Even with a topic as narrowly defined as SGM cancer health equity research, this conference succeeded in bringing people together, highlighting the current state of the science, and gathering feedback for future research studies.

## Data Availability

Data on conference satisfaction and feedback is available upon request to the corresponding author.

## Abbreviations

SGM: Sexual and Gender Minorities
SOGI: Sexual Orientation and Gender Identity
